# Enrollment of underrepresented racial and ethnic groups in the Rare and Atypical Diabetes Network (RADIANT)

**DOI:** 10.1017/cts.2022.529

**Published:** 2023-01-23

**Authors:** Mustafa Tosur, Laura Gandolfo, Ashok Balasubramanyam, Rochelle N. Naylor, Toni I. Pollin, Neda Rasouli, Sara J. Cromer, John B. Buse, Maria J. Redondo, RADIANT Study Group

**Affiliations:** 1 Department of Pediatrics, The Division of Diabetes and Endocrinology, Baylor College of Medicine, Texas Children’s Hospital, Houston, TX, USA; 2 Children’s Nutrition Research Center, USDA/ARS, Houston, TX, USA; 3 Health Informatics Institute, University of South Florida, Tampa, FL, USA; 4 Division of Diabetes, Endocrinology and Metabolism, Baylor College of Medicine, Houston, TX, USA; 5 Division of Adult and Pediatric Endocrinology Diabetes, and Metabolism, Departments of Pediatrics and Medicine, University of Chicago, Chicago, IL, USA; 6 Departments of Medicine and Epidemiology & Public Health, University of Maryland, Baltimore, MD, USA; 7 Division of Endocrinology, Metabolism and Diabetes, Department of Medicine, University of Colorado, Aurora, CO, USA; 8 Division of Endocrinology, Diabetes and Metabolism, Department of Medicine, Massachusetts General Hospital, Boston, MA, USA; 9 Division of Endocrinology, Department of Medicine, University of North Carolina, Chapel Hill, NC, USA

**Keywords:** Race, ethnicity, hispanic ethnicity, African American race, clinical research

## Abstract

**Introduction:**

Diabetes mellitus in underrepresented racial and ethnic groups (URG) is rapidly increasing in incidence and has worse outcomes than diabetes in non-Hispanic White individuals. Rare and Atypical Diabetes Network (RADIANT) established recruitment targets based on the racial and ethnic distribution of the USA to enroll a diverse study population. We examined participation of URG across RADIANT study stages and described strategies to enhance recruitment and retention of URG.

**Materials and Methods:**

RADIANT is a multicenter NIH-funded study of people with uncharacterized forms of atypical diabetes. RADIANT participants consent online and progress through three sequential study stages, as eligible.

**Results:**

We enrolled 601 participants with mean age 44 ± 16.8 years, 64.4% female. At Stage 1, 80.6% were White, 7.2% African American (AA), 12.2% other/more than one race, and 8.4% Hispanic. Enrollment of URG was significantly below preset targets across most stages. Referral sources differed by race (*p* < 0.001) but not ethnicity (*p* = 0.15). Most AA participants were referred by RADIANT investigators (58.5% vs. 24.5% in Whites), whereas flyers, news, social media, and family or friends were more frequent referral sources for White individuals (26.4% vs. 12.2% in AA). Ongoing initiatives to increase enrollment of URG in RADIANT include engaging with clinics/hospitals serving URG, screening electronic medical records, and providing culturally competent study coordination and targeted advertisement.

**Conclusions:**

There is low participation of URG in RADIANT, potentially limiting the generalizability of its discoveries. Investigations into barriers and facilitators for recruitment and retention of URG in RADIANT, with implications for other studies, are ongoing.

## Introduction

Diabetes mellitus is understudied in underrepresented racial and ethnic groups (URG). URG with diabetes tend to have younger age at onset and worse outcomes [1–3] and are experiencing a more rapid increase in the incidence of this disease [4,5]. Also, distinct biological differences in diabetes pathophysiology [6–9] between ancestries underscore the critical importance of studying population diversity for the advancement of the field. Historically, there have been large gaps in participation rates of various racial and ethnic groups in clinical research [10], particularly in studies involving genetics or genetic tests. Although some of the reasons are known, including medical and research mistreatment resulting in distrust, poor access to health care and other barriers [11], recruitment strategies that could enhance participation of URG in clinical research remain to be elucidated. Lack of diversity of research participants remains a critical concern in clinical research and for equity in medical advances.

The Rare and Atypical Diabetes Network (RADIANT) is an NIH/NIDDK-supported study to identify and study individuals with rare and uncharacterized forms of diabetes in the USA. Persons referred to RADIANT provide online consent upon enrollment and complete a screening questionnaire to determine if they are eligible for study participation. They may then progress through three sequential study stages, depending on data obtained in each preceding stage. Study procedures include genomic, transcriptomic, and metabolomic analyses. In the absence of extant data on racial/ethnic distributions of atypical forms of diabetes, recruitment targets by race and ethnicity for RADIANT were set based on racial/ethnic distribution of the US population.

We hypothesized that enrollment of URG in an NIH-funded multicenter consortium would be challenging despite focused efforts for enrollment of participants from diverse racial and ethnic backgrounds. We aimed to study the racial/ethnic distribution of current RADIANT participants in comparison with the targeted racial/ethnic distribution and to describe ongoing and planned recruitment strategies to improve research participation of URG.

## Materials and Methods

### Participants

At the time of this data collection, RADIANT consisted of 14 study clinics with one data coordinating center. Individuals with suspected atypical diabetes were referred to RADIANT from the study clinical sites, existing atypical diabetes registries, established diabetes cohorts, prospective clinic registries, and non-RADIANT clinical providers. Electronic medical record screening methods were also employed to identify and subsequently contact persons with potentially atypical phenotypes of diabetes. In addition, individuals could self-refer. Between September 2020 and April 2022, RADIANT enrolled 601 adult and pediatric participants with suspected atypical diabetes. A participant was suspected to have “atypical diabetes” if he/she had a diagnosis of diabetes mellitus and met one of the RADIANT criteria for atypicality (e.g., type 2 diabetes diagnosed when the individual was prepubertal or non-obese, being lean and insulin-resistant, etc.). The following categories of patients were excluded from study participation: those with high likelihood of “typical” type 1 or type 2, known monogenic, or other known secondary forms of diabetes; those declining to consent for genetic testing, and current pregnancy. All study participants provided informed consent prior to screening and enrollment, and the study was approved by a central Institutional Review Board (IRB) at the University of Utah, with IRBs at each clinical site approving the reliance on the central IRB as the IRB of record.

### Procedures

**Fig. 1.**
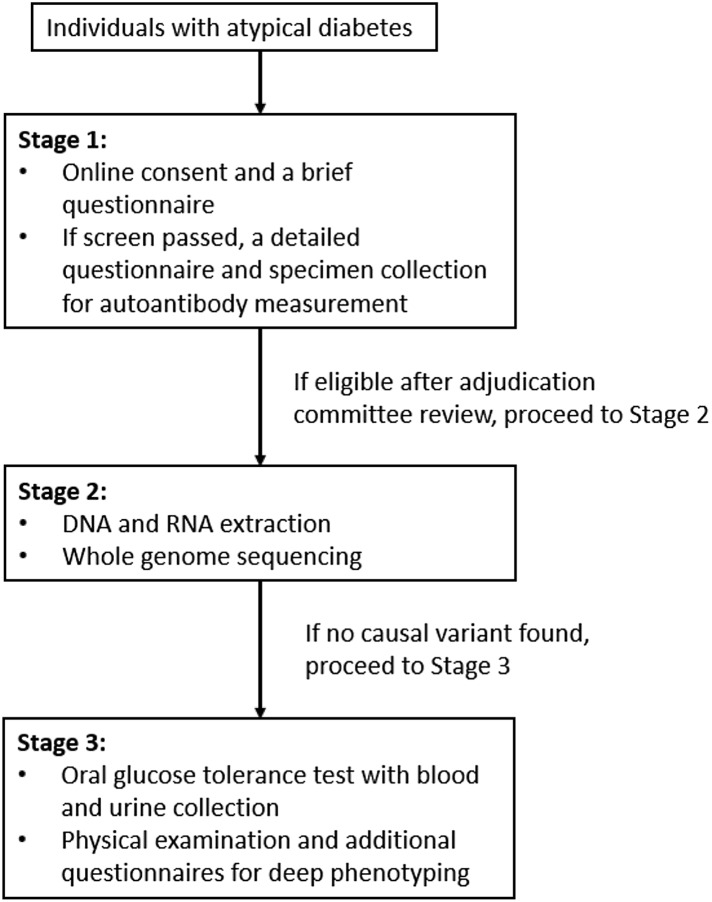
RADIANT study stages. RADIANT, Rare and Atypical Diabetes Network.

The study protocol has three sequential stages (Fig. 1). In Stage 1, individuals complete an online consent form and a brief questionnaire to exclude the likelihood that they have a typical form of type 1 or type 2 diabetes, a known monogenic form of diabetes or a known form of secondary diabetes. If they do not meet these exclusion criteria, participants are deemed “screen pass” and asked to complete a detailed online Stage 1 questionnaire and to undergo blood collection for islet autoantibody testing at a local laboratory or a clinical site. The information on the referral source for each participant is collected via one of the sections of this Stage 1 questionnaire (Supplementary Material). Each case is then reviewed by an Adjudication Committee, a panel of expert endocrinologists, geneticists, and diabetes researchers who examine the questionnaire data, any additional information from the participant or their physician and the autoantibody results to select candidates to proceed to Stage 2. In Stage 2, participants re-consent to undergo another blood collection for DNA and RNA extraction, with whole genome sequencing is performed and evaluated in a clinical laboratory. The participant then proceeds to Stage 3. In Stage 3, participants undergo a detailed physical exam, an oral glucose tolerance test, and additional blood and urine collection in one of the study clinical sites for deeper phenotyping. Accumulated data are then reviewed and discussed by the Discovery Team – a group of experts in clinical and molecular genetics, bioinformatics, clinical diabetes, and metabolic phenotyping, to categorize the potential form of atypical diabetes, determine whether family members should be enrolled to support a potential novel genetic diagnosis, and recommend additional phenotyping tests. At study design, it was anticipated that 50% of Stage 1 enrollees would progress to Stage 2, and approximately 90% of Stage 2 enrollees to Stage 3.

Overall enrollment rate and enrollment of URG are reviewed regularly by the RADIANT Recruitment and Retention Committee. Enrollment of URG has been one of the key focus areas of this committee, which also compiled data on different initiatives undertaken at RADIANT sites to improve participation of URG via survey sent to the sites.

### Race and Ethnicity Categorization

We determined race and ethnicity categorizations based on self-report and standard NIH classifications for race and ethnicity [12]. In this study, we assessed recruitment by ethnicity (Hispanic vs. non-Hispanic) and by the following four racial categories: 1) American Indian or Alaska Native/Asian/Native Hawaiian or Other Pacific Islander; 2) Black or African American (AA); 3) White; and 4) more than one race.

### Statistical Analyses

Baseline characteristics of those screened were summarized. For each study stage milestone, the one sample test of proportions was used to measure differences between observed ethnicity and race proportions and preset target enrollment proportions. Based on expected cell size, the Chi-square test or Fisher’s exact test was used to measure differences in the observed race and ethnicity proportions, and referral sources. All analyses were performed using SAS version 9.4 (Cary, NC). Using Bonferroni correction for multiple testing, *p*-values < 0.005 were considered statistically significant (number of comparisons = 10).

## Results

Six hundred and one participants met the RADIANT inclusion and exclusion criteria and were enrolled in Stage 1. The mean age at study participation was 44 ± 16.8 years, with 64.4% female. Among participants with available race/ethnicity data, 80.6% were White, 7.2% AA, 12.2% other race/ethnicity, and 8.4% Hispanic. Race and ethnicity data were not available for 33 (5.5%) and 16 (2.7%) participants, respectively. Other participant characteristics are shown in Table 1.

**Table 1.** Baseline characteristics of subjects at study participation (*n* = 601)

	Missing (*n*)	Mean ± SD or *n* (%)
Age at enrollment, years	1	44 ± 16.8
Age at diabetes diagnosis, years	8	31.5 ± 15.5
Female, *n* (%)	6	383 (64.4%)
Hispanic, *n* (%)	16	49 (8.4%)
Race, *n* (%) American Indian/Alaska Native/Asian/Native Hawaiian or other Pacific Islander Black or African American White More than one race	33	46 (8.1%) 41 (7.2%) 458 (80.6%) 23 (4.1%)
BMI at enrollment, kg/m^2^	35	25.5 ± 6
BMI at diabetes diagnosis, kg/m^2^	129	25 ± 6.8
HbA1c at diagnosis, %	298	9.6 ± 3.2
Most recent HbA1c, %	395	6.9 ± 1.7
Ever taken insulin, *n* (%)	255	260 (75.1%)
Currently taking insulin, *n* (%)	343	223 (86.4%)
Islet autoantibody positivity, *n* (%)	229	73 (19.6%)
Family history of diabetes or hyperglycemia, *n* (%)	89	418 (81.6%)

Unless otherwise noted, mean ± SD was provided for each variable.

The enrollment rate for URG was significantly below preset targets at consent and across most stages (Table 2). Preset target enrollment rates were 16.5% and 15.5% for Hispanic and AA participants, respectively, for each stage. In comparison, among participants who completed the Stage 1 questionnaire, 8.4% were Hispanic and 7.2% AA (both *p* < 0.001). The racial/ethnic distribution of those completing the blood collection for islet autoantibody measurement in Stage 1 was similar to the percentages in the preceding step (8.4% and 6.3% in Hispanic and AA participants, respectively). Among participants eligible for Stage 2 and Stage 3, the percentages of Hispanic participants further decreased to 7% and 4.2%, respectively (compared with preset rate of 16.5%, *p* < 0.001 and *p* = 0.001, respectively). Similarly, the percentages of AA race among participants eligible for Stage 2 and Stage 3 were 8.3% (*p* = 0.008) and 4.2% (*p* = 0.004), respectively, compared with the preset rate of 15.5%.

**Table 2.** Comparison of the target enrollment percentage for races/ethnicities and the current participation percentages in different stages of RADIANT

		Participants in Stage 1				Participants eligible for:			
		Consented, passed screening, and completed Stage 1 questionnaire (*n* = 601)		Specimen return (*n* = 375)		Stage 2 (*n* = 173)		Stage 3 (*n* = 97)	
Demographic	Target %	*N* (%)	*p*-Value	*N* (%)	*p*-Value	N (%)	*p*-Value	N (%)	*p*-Value
Hispanic/Latino	16.5%	49 (8.4%)	**<0.001**	31 (8.4%)	**<0.001**	12 (7%)	**<0.001**	4 (4.2%)	**0.001**
Not Hispanic/Latino	83.5%	536 (91.6%)		339 (91.6%)		160 (93%)		92 (95.8%)	
Missing ethnicity data		16		5		1		1	
American Indian/Alaska Native/Asian/Native Hawaiian or Other Pacific Islander	10%	46 (8.1%)	**<0.001**	31 (8.5%)	**<0.001**	20 (11.9%)	0.008	13 (13.5%)	**0.004**
Black or African American	15.5%	41 (7.2%)		23 (6.3%)		14 (8.3%)		4 (4.2%)	
White	65%	458 (80.6%)		294 (81%)		126 (74.6%)		74 (77.1%)	
More than one race	9.5%	23 (4%)		15 (4.1%)		9 (5.2%)		5 (5.2%)	
Missing race data		33		12		4		1	

RADIANT, Rare and Atypical Diabetes Network.

We investigated for differences in referral sources between racial/ethnic groups. The referral sources differed significantly by race (*p* < 0.001) but not ethnicity (*p* = 0.15) (Table 3). Most AA participants (58.5%) were referred by RADIANT investigators (vs. 24.5% of White participants). In contrast, the largest percentage of White participants (26.4%) were self-referred, having learned about the study from flyers, news media, social media, and family or friends, whereas these sources accounted for only 12.2% of AA participants.

**Table 3.** Comparison of referral sources between racial/ethnic groups

	Any source	RADIANT site or doctor	Non-RADIANT doctor or provider	Studies or registries	Flyer, news report, social media, family, or friend	Other or unknown source	*p*-Value
Hispanic/Latino	49	20 (40.8%)	10 (20.4%)	3 (6.1%)	5 (10.2%)	11 (22.4%)	0.15
Non-Hispanic/Latino	536	156 (29.1%)	102 (19.0%)	30 (5.6%)	134 (25.0%)	114 (21.3%)	
American Indian/Alaska Native/Asian/Native Hawaiian, or Other Pacific Islander	46	21 (45.7%)	8 (17.4%)	3 (6.5%)	9 (19.6%)	5 (10.9%)	**<0.001**
Black or African American	41	24 (58.5%)	7 (17.1%)	3 (7.3%)	5 (12.2%)	2 (4.9%)	
White	458	112 (24.5%)	90 (19.7%)	24 (5.2%)	121 (26.4%)	111 (24.2%)	
More than one race	23	9 (39.1%)	4 (17.4%)	2 (8.7%)	3 (13.0%)	5 (21.7%)	

RADIANT, Rare and Atypical Diabetes Network.

Efforts undertaken by RADIANT sites to enhance URG participation in the different study stages were investigated (Table 4). Several sites are actively engaged with extramural physicians and/or clinics serving URG to facilitate patient referrals to RADIANT. In addition, many sites are working with local affiliated clinics, hospitals, and nonprofit organizations working with populations from URG. Some sites developed and implemented screening approaches using electronic medical record systems to identify individuals with atypical diabetes in an unbiased manner, who were then approached by study staff to present the study and encourage participation. After identifying language as a barrier to initial screening and/or progression through the RADIANT study stages, culturally and linguistically competent study coordination was provided in many sites by making a bilingual research coordinator available to help with enrollment of Spanish-speaking individuals. Site staff helped participants navigate and complete study procedures to help mitigate retention barriers due to limited health literacy or access. Existing studies and registries were leveraged to identify subjects with atypical diabetes for referral to RADIANT including the Monogenic Diabetes Registry at the University of Chicago [13], the Personalized Diabetes Medicine Program at the University of Maryland [14], and the Ketosis-Prone Diabetes Registry at Baylor College of Medicine [15]. Multiple advertisement tools and avenues were used to enhance awareness of RADIANT in the community, among clinicians and potential participants. Although the target enrollment percentages for URG are not met fully in RADIANT as of this report, a substantial improvement has been made in the participation of URG by leveraging these various initiatives (Table 5).

**Table 4.** Summary of strategies to facilitate recruitment of underrepresented racial and ethnic groups (URG) by RADIANT sites

	Recruitment Strategies
**1**	Engaging with physicians/clinics serving URG
**2**	Working with local organizations, including, but not limited to local affiliated clinics/hospitals, local affiliated nonprofit organizations/companies/groups
**3**	Implementing electronic medical record screening approaches to identify patients with atypical diabetes for RADIANT in an unbiased manner
**4**	Utilizing culturally competent study coordination to help focus on URG recruitment efforts (e.g., Spanish-speaking coordinators, etc.)
**5**	Utilizing on-site resources/programs for engagement and community connections
**6**	Targeted advertising and utilizing existing RADIANT advertisement tools (i.e., flyers, emails, radio stations)
**7**	Connecting with existing studies and registries
**8**	Utilizing a Clinical and Translational Science Awards (CTSA)-Funded Institute/Program
**9**	Increased site ability to assist potential participants with Stage 1 consent, questionnaires, and specimen collection

URG, underrepresented racial and ethnic groups; RADIANT, Rare and Atypical Diabetes Network.

**Table 5.** The percentages of URG among Step 1 eligible participants in RADIANT since the study launch (Q4 – 2020)

All Stage 1 eligible (consented, passed screening, and completed Stage 1 questionnaire)		Non-White (multi-racial, Black, American Indian or Alaska Native, Asian, Native Hawaiian, or Other Pacific Islander)		Black		American Indian or Alaska Native, Asian, Native Hawaiian, or Other Pacific Islander		Hispanic	
	**N**	**N**	**% of All**	**N**	**% of All**	**N**	**% of All**	**N**	**% of All**
Q4 2020	217	31	14.3%	10	4.6%	12	5.5%	16	7.4%
Q1 2021	134	20	14.9%	4	3.0%	14	10.4%	9	6.7%
Q2 2021	68	19	27.9%	4	5.9%	8	11.8%	8	11.8%
Q3 2021	49	10	20.4%	7	14.3%	2	4.1%	5	10.2%
Q4 2021	72	14	19.4%	10	13.9%	3	4.2%	6	8.3%
Q1 2022	61	16	26.2%	6	9.8%	7	11.5%	5	8.2%
*Total*	*601*	*110*		*41*		*46*		*49*	

URG, underrepresented racial and ethnic groups; RADIANT, Rare and Atypical Diabetes Network.

## Discussion

We set out to investigate participation of URG in RADIANT, a national consortium to study rare and atypical diabetes. Participation of URG was significantly below preset targets across study stages despite multiple initiatives to reach people of Hispanic ethnicity and AA race. Referral sources differed significantly by race but not by ethnicity.

Since the publication of NIH guidelines mandating inclusion of women and minorities in clinical research in 1993, engagement of URG in research has received increased attention by the scientific community. At study participation in Stage 1, the percentages of Hispanic and AA participants were half and one-third, respectively, of preset targets, revealing significant racial and ethnic disparities in participation in our study. Similar to our findings and others [16–18], a recent report on participation in phase 3 randomized cancer trials based on data from ClinicalTrials.gov showed overrepresentation of White participants and underrepresentation of AA and Hispanic participants [19]. Although some studies report lower levels of willingness by AA and Hispanic individuals to participate in biomedical research, or studies involving invasive procedures or genetic tests [20,21], other studies show no significant difference between AA, Hispanic, and non-Hispanic White individuals in participation rates [22,23].

Having sufficient URG participation in clinical research is of critical importance to health advances and health equity. Generalizability of research findings to large, multiethnic populations requires appropriate representation of the constituent racial and ethnic groups. Several diabetes studies have highlighted significant racial and ethnic differences in natural history [3,5–7,9], prognosis [1,2,24], and treatment responses [25]. Better understanding of these differences and their implications may help us address existing racial and ethnic health disparities.

Multiple barriers and facilitators to participation of URG in biomedical research have been identified [20,26,27]. Common barriers include lack of trust, cultural barriers, competing demands, language barrier, study burden (i.e., study duration, follow-up visits, and procedures), expenses associated with travel and/or missed work, lack of health insurance coverage, and low health literacy. A legacy of concerns about abuse appeared as one of the leading barriers particularly for AA individuals, in a systematic review analyzing data from 44 articles [26]. Memories of the Tuskegee study in which uninformed AA men were enrolled in a longitudinal observational study to understand the natural history of syphilis and not provided treatment with penicillin even after the efficacy of the drug was established, have lasting effects on the attitudes of many AA individuals toward clinical research [28]. Much foundational work related to re-establishing trust and ensuring transparency and research integrity is needed to regain the full confidence of the AA community in the clinical research process. On the other side, facilitators include cultural congruence between research staff and participants, monetary compensation for time spent completing research procedures, access to medical services through research, altruism, low risk and/or burden of participation, and convenience. Addressing mistrust by assuring safety, developing trust between researchers and participants, providing a range of treatment options and/or enrolling from diverse racial/ethnic groups emerged as important facilitators, particularly for the AA community [26]. The observation that the predominant referral source to RADIANT for AA participants is RADIANT investigators highlights the critical importance of gaining the trust of potential participants through patient-provider and other direct personal interactions.

URG may have lower access to sources of reliable information about research [17,18] and clinical care [29] than White populations, which may lead to lower self-referral to RADIANT or decreased ability to complete the enrollment protocol after referral by their clinician. In addition, lack of familiarity by clinicians with atypical diabetes in racial/ethnic minorities may lead to lower referral to RADIANT. To overcome these potential barriers to enrollment of URG and enhance their participation in RADIANT, recruitment strategies as described in Table 4 were implemented by RADIANT sites. Many of these initiatives focus on either utilizing a culturally sensitive recruitment approach and developing trust or facilitating a referral process from sources with whom potential participants have an established relationship. It is important to note that despite all the initiatives implemented, enrollment rates for URG are currently below targeted goals, warranting further investigation to develop new initiatives to support URG participation.

A potential limitation of our study includes using a self-reported race and ethnicity categorization. However, self-reported race/ethnicity data have been shown to be highly concordant with genetic ancestry using genome-wide genotypes and principal component analyses [30]. Moreover, the emphasis on diversity is not solely to assure ancestral representation but also importantly to address systematic inequities based on social determinants of health. Although the sample size of some groups (i.e., American Indians, Alaska Natives, Asians, Native Hawaiians, and other Pacific Islanders) was insufficient for meaningful analyses, the number of AA and Hispanic participants, enrolled in a national observation study from a wide geographic area in the USA, allowed us to study differences in the rate of participation for these groups. Another limitation of our study is the lack of data on the prevalence of atypical diabetes among individual racial/ethnic groups making reliable target determination challenging. Further studies are warranted to assess the effectiveness of each initiative as this was beyond the scope of our current study and practically was not possible due to simultaneous implementation of multiple initiatives.

In conclusion, participation of URG in RADIANT to date is suboptimal. Recognition of this long-standing issue in diabetes-related clinical research could be an important initial step toward improving URG participation. Further understanding of the barriers and facilitators in RADIANT should enhance URG participation in RADIANT and provide a roadmap for other human studies. Increased recognition of disparities in diabetes clinical research participation and awareness of potential strategies to address this gap may facilitate equitable participation and benefit from studies leading to accelerated scientific discoveries and improved disease outcomes.

## Supplementary material

For supplementary material accompanying this paper visit https://doi.org/10.1017/cts.2022.529. click here to view supplementary material

## Prior Presentation

Part of this study was presented at the 82^nd^ Scientific Sessions of the American Diabetes Association, June 3–7, 2022 (New Orleans, LA).
