# Supplementary material for: Enrollment of underrepresented racial and ethnic groups in the Rare and Atypical Diabetes Network (RADIANT)
Source: J Clin Transl Sci. 2023 Jan 23;7(1):e47. doi: 10.1017/cts.2022.529 (PMC9947614; doi:10.1017/cts.2022.529)
Supplement: Supplementary file 1 [file ctssup.zip › S2059866122005295sup001.docx]

**RADIANT Questionnaire Section 1**

Please answer the questions below about your diabetes diagnosis, medical history, and family history and click “Submit” when you are finished. This information will help us determine if you qualify for further participation in the RADIANT study.

*Note: If you are completing this questionnaire on behalf of the study participant, “you” means “the study participant”.*

| 1. Are you pregnant now?   *Individuals who are pregnant are not able to participate in RADIANT.*   - *If you are pregnant now: You will not be able to participate in the study now. If you are otherwise eligible for the study, you may participate in the study after your pregnancy.* - *If you are not pregnant now or are not sure: You may participate in the study at this time, if you meet the other eligibility criteria based on your responses in this questionnaire section. If you become pregnant while in the study, please let the RADIANT study team know; you will need to stop your study participation while pregnant.* | ⭘Yes ⭘No |
| --- | --- |
| 1. Have you been diagnosed with diabetes? | ⭘Yes ⭘No ⭘Don’t Know |
| 1. Do you take diabetes medications (e.g., insulin, metformin, other)? | ⭘Yes ⭘No ⭘Don’t Know |
| 1. Have you ever had diabetic ketoacidosis (DKA)? | ⭘Yes ⭘No ⭘Don’t Know |
| 1. Did you have DKA at the time your diabetes was diagnosed? | ⭘Yes ⭘No ⭘Don’t Know |
| 1. What age were you when you were diagnosed with diabetes (years)? |  |
| 1. Do you remember the date you were diagnosed with diabetes? | ⭘Yes ⭘No ⭘Don’t Know |
| - 1. Please enter the date you were diagnosed with diabetes (MM-DD-YYYY) |  |
| 1. Have you been continuously treated with insulin since diagnosis (i.e., you have not been off insulin for longer than one week)? | ⭘Yes ⭘No ⭘Don’t Know |
| 1. Have you ever been tested in the past for diabetes autoantibodies? Autoantibody examples: GAD65, ICA, IA2/ICA512, Insulin/IAA, ZnT8. | ⭘Yes ⭘No ⭘Don’t Know |
| - 1. What was the result of the antibody testing? | ⭘ Positive: I had antibodies (typically consistent with type 1 diabetes)  ⭘ Negative: I did not have antibodies  ⭘ Don’t Know |
| 1. Was your diabetes diagnosed during pregnancy? | ⭘Yes ⭘No ⭘Don’t Know |
| - 1. Did the diabetes stay, or come back at any time, after you were pregnant? | ⭘Yes ⭘No ⭘Don’t Know |
| 1. Were you on corticosteroids (e.g., prednisone, hydrocortisone, dexamethasone, etc.) by mouth or injection for a month or longer at the time when you were diagnosed with diabetes? | ⭘Yes ⭘No ⭘Don’t Know |
| - 1. Did the high blood sugars continue or come back after steroid treatment was stopped? | ⭘Yes ⭘No ⭘Don’t Know |
| 1. Did you have a pancreatectomy (surgery to remove all or most of the pancreas) before you were diagnosed with diabetes? | ⭘Yes ⭘No ⭘Don’t Know |
| 1. Did you have chronic pancreatitis (repeated episodes of the pancreas getting inflamed) before you were diagnosed with diabetes? | ⭘Yes ⭘No ⭘Don’t Know |
| 1. Were you undergoing chemotherapy before you were diagnosed with diabetes? | ⭘Yes ⭘No ⭘Don’t Know |
| 1. Did you have HIV or take medications for HIV before you were diagnosed with diabetes? | ⭘Yes ⭘No ⭘Don’t Know |
| 1. Do you have cystic fibrosis? | ⭘Yes ⭘No ⭘Don’t Know |
| 1. Do you have hemochromatosis (a condition where too much iron accumulates in blood)? | ⭘Yes ⭘No ⭘Don’t Know |
| 1. Do you have Cushing’s syndrome (too much cortisol in the body)? | ⭘Yes ⭘No ⭘Don’t Know |
| 1. Do you have acromegaly (too much growth hormone in the body)? | ⭘Yes ⭘No ⭘Don’t Know |
| 1. Do you have alternating periods of both high blood sugars (hyperglycemia) and low blood sugars (hypoglycemia) when you are not taking diabetes medications? | ⭘Yes ⭘No ⭘Don’t Know |
| 1. How many biological parents, siblings, or children **with diabetes** do you have?   *‘Biological’ means family members related to you by blood* | ⭘ None  ⭘ One  ⭘ Two  ⭘ Three or more  ⭘ Don’t Know |
| 1. Have you been told by a physician that your diabetes is atypical, rare or cannot be classified? | ⭘Yes ⭘No ⭘Don’t Know |
| 1. Which physician told you this? | [Select from drop-down list] |
| 1. Please describe what they said about your diabetes: |  |
| 1. Why do you think that you qualify for this study on atypical or rare cases of diabetes? Is there any additional information (ex. additional medical history information, unique diagnosis experience) not captured in your previous responses that you think makes you a good fit for this study? |  |
| 1. How did you hear about RADIANT? (Check all that apply) | ⭘ My doctor or diabetes care provider told me about this study  ⭘ I received a letter telling me about this study  ⭘ I heard through my involvement in an existing research study  ⭘ I saw a news report  ⭘ I saw a RADIANT Study flyer posted on social media (Facebook, Twitter, etc)  ⭘ I saw a RADIANT study flyer posted near my doctor’s office or near where I live  ⭘ Through a friend or family member  ⭘ Other  ⭘ Don’t Know |
| 1. Please list the name of the doctor or diabetes care provider who told you about RADIANT: |  |
| 1. Please select which research study: | ⭘ University of Chicago Monogenic Diabetes Registry or other University of Chicago study  ⭘ University of Maryland Personalized Diabetes Medicine Program (PDMP)  ⭘ University of Michigan Lipodystrophy Tissue and Blood Biorepository study or the Natural History Study (LD-Lync)  ⭘ Washington University Wolfram Registry  ⭘ Baylor College of Medicine Ketosis Prone Diabetes Registry  ⭘ SEARCH for Diabetes in Youth  ⭘ TODAY study  ⭘ Other study  ⭘ Don’t Know |
| 1. Please specify the other research study: |  |
| 1. Please describe how you heard about RADIANT: |  |
| 1. Were you referred by a RADIANT Study Clinical Site? | ⭘Yes ⭘No ⭘Don’t Know |
| 1. Please select which RADIANT Study Clinical Site: | ⭘ Baylor College of Medicine  ⭘ Columbia University  ⭘ Indiana University  ⭘ Massachusetts General Hospital  ⭘ Seattle Children’s  ⭘ SUNY Downstate Health Science University  ⭘ University of Chicago  ⭘ University of Colorado  ⭘ University of Maryland  ⭘ University of Michigan  ⭘ University of North Carolina  ⭘ University of Washington  ⭘ Vanderbilt University  ⭘ Washington University |
| 1. Are you related to someone else who is participating in the RADIANT study? | ⭘ Yes ⭘ No ⭘ Don’t Know |
| - 1. Did the RADIANT team give you an enrollment code to enter in this questionnaire?   *(It is okay if you did not receive a code. Only some family members will receive a code to enter here, depending on how they were referred to RADIANT.)* | ⭘ Yes ⭘ No ⭘ Don’t Know |
| - - 1. Please enter the code: | _________________ |

When you are finished, please click “Submit”.

[Submit]
