# Supplementary material for: Enrollment of underrepresented racial and ethnic groups in the Rare and Atypical Diabetes Network (RADIANT)
Source: J Clin Transl Sci. 2023 Jan 23;7(1):e47. doi: 10.1017/cts.2022.529 (PMC9947614; doi:10.1017/cts.2022.529)
Supplement: Supplementary file 1 [file ctssup.zip › S2059866122005295sup002.docx]

**The RADIANT Study Group**

**Baylor College of Medicine:** Ashok Balasubramanyam, M.D., PI^1,2,3,4,5,6,7,8,9^, Maria J. Redondo, M.D., Ph.D., M.P.H., PI^1,3,6,7,8^, Mary Ann Fang, Marcela Astudillo, M.D., Ansley Davis, Dimpi Desai, M.D., Ruchi Gaba, M.D., Nupur Kikani, M.D., Elizabeth Kubota-Mishra, M.S., D.O., Narayan Mulukutla, M.D., Nikalina G O’Brien, Jennifer Posey, M.D., Ph.D. ^1,3,7,9^, Stephanie Sisley, M.D.^3^, Mustafa Tosur, M.D.^1^ *Past Staff: Adriana Cardenas, Adrienne Ideozu, Erica Hattery, M.D., M.H.A., Julizza Jimenez, Graciela Montes, Lee-Jun Wong, Ph.D.*

**Columbia University:** Robin Goland, M.D., PI^1,3,4,8^, Wendy Chung, M.D., Ph.D.^7^, Rachelle Gandica, M.D., Rudolph Leibel, M.D.^3^, James Pring

**Indiana University:** Carmella Evans-Molina, Ph.D., M.D., PI^1,3,7^, Gabriela Monaco, M.D., Anna Neyman, M.D., Zeb Saeed, M.D., Emily Sims, M.D., Maria Spall. *Past Staff: Marimar Hernandez-Perez, Ph.D., Kelly Moors*

**Massachusetts General Hospital:** Miriam S. Udler, M.D., Ph.D., PI^1,3,7,8^, Jose C. Florez, M.D., Ph.D., PI^1,2,3,4,5,6,7,8,9^, Melissa Calverley, Victoria Chen, Kathy Chu, Sara Cromer, M.D., Aaron Deutsch, M.D., Mariella Faciebene, Evelyn Greaux^6^, Dorit Koren, M.D., Raymond Kreienkamp, M.D., Ph.D., Mary Larkin, R.N., M.S., CDCES, Pam Ricevuto, Amy Sabean, R.N., Jordan Sherwood, M.D., Nopporn Thangthaeng R.N., Ph.D., CDCES

**NorthShore University HealthSystem:** Liana K. Billings, M.D., M.M.Sc., PI^1,3^

**SUNY Downstate Health Sciences University:** Mary Ann Banerji, M.D., PI^1,3^, Necole Brown, Lina Soni, M.D., Lorraine Thomas.

**University of Chicago:** **University of Chicago:** Louis H. Philipson, M.D., Ph.D., PI^1,2,3,4,5,6,7,8,9^, Siri Atma W. Greeley, M.D., Ph.D., PI^1,3^, Marilyn Arosemena, M.D., Graeme Bell, Ph.D.^3,7^, Colleen Bender, Shanna Banogon, Jui Desai, David Ehrmann, M.D.^3,5,6,7^, Lisa R. Letourneau-Freiberg, M.P.H.^5,6,8^, Rochelle N. Naylor, M.D.^1,3,7,8^, Forough Noohi, Lainie Friedman Ross, M.D., Ph.D.^3,5,6^, Maria Salguero-Bermonth, M.D., Erin Wright.

**University of Colorado – Denver:** Neda Rasouli, M.D., PI^1,3,7,8^, Chelsea Baker, Noosha Farhat, Andrew Her, Courtney King. *Past Staff: Jules Barklow, Rebecca Lorch, Carter Odean, M.S., Gregory Schleis, M.D., Chantal Underkofler*

**University of Maryland:** Toni I. Pollin, Ph.D., M.S., PI^1,3,8^, Kristin Maloney, M.S., M.G.C.^3^, Ryan Miller, M.D., Paula Newton, M.D., Maria Eleni Nikita, M.D., Devon Nwaba, M.P.H.^5,6^, Kathleen Palmer, Stephanie Riley M.S., Kristi Silver, M.D., Hilary Whitlatch, M.D.^1^ *Past Staff: Elizabeth Streeten, M.D.*

**University of Michigan:** Elif Oral, M.D., PI^1,3,5,7,8^, Maria Foss de Freitas, M.D., Brigid Gregg, M.D., Seda Grigoryan, M.D., Melda Sonmez Ince, M.D., Adam Neidert, M.S., Carman Richison. *Past Staff: Baris Akinci, M.D., Rita Hench*

**University of North Carolina:** John Buse, M.D., Ph.D., PI^8^, Jamie Diner, M.S.N.^3,8^, Karthik Edupuganti, Rachael Fraser, Karla Fulghum, Alex Kass^5,6^, Klara Klein, M.D., Ph.D.^1^, Carlos Velez. *Past Staff: M. Sue Kirkman, M.D.*

**University of Washington:** Irl B. Hirsch, M.D., PI^1,3^, Jesica Baran, M.D., Xiaofu Dong, Steven Kahn, M.D.^1,3^, Thanmai Kaleru, Dori Khakpour^6^, Lori Sameshima

**Seattle Children's:** Catherine Pihoker, M.D., PI^1,3,5,6,7^, Beth Loots, M.P.H., M.S.W.^6^ *Past Staff: Cisco Pascual*

**Vanderbilt University:** Kevin Niswender, M.D., Ph.D., PI, Justin Gregory, M.D., M.S.C.I., Alvin Powers, M.D.^1,3^, Andrea Ramirez, M.D., M.S.C.I.^1,3^, Jordan Smith^6^. *Past Staff: Jennifer Scott*

**Washington University:** Fumihiko Urano, M.D., Ph.D., PI^1,3,7^, Jing Hughes, M.D., Ph.D.^1,3,7^, Stacy Hurst, Jennifer May, M.D., Janet McGill, M.D., M.A.^1,3,5,6,8^, Stephen Stone, M.D.^1,3,7^

**Data Coordinating Center – University of South Florida:** Jeffrey P. Krischer, Ph.D., PI^1,2,3,4,5,6,7,8,9^, Rajesh Adusumalli, M.S., Bruce Albritton, Analia Aquino, Paul Bransford, Nicholas Cadigan, Laura Gandolfo, Jennifer Garmeson, Joseph Gomes, M.S.Cp.E, Robert Gowing, Christina Karges, M.P.H., Callyn Kirk, M.S.P.H., Sarah Muller^4^, Jean Morissette, Hemang M. Parikh, Ph.D.^8^, Francisco Perez-Laras, Cassandra L. Remedios, M.S.^7,8^, Pablo Ruiz, Noah Sulman, Ph.D., Michael Toth, M.S.H.I., Lili Wurmser. *Past Staff: Christopher Eberhard, M.S., Steven Fiske, Brandy Hutchinson, C.I.P., Sidhvi Nekkanti,* *Rebecca Wood, M.S.*

**Genetics Core – Broad Institute:** Jose C. Florez, M.D., Ph.D., PI^1,2,3,4,5,6,7,8,9^, Ahmed Alkanaq, Ph.D.^3^, MacKenzie Brandes, Nöel Burtt, Jason Flannick, Ph.D.^3^, Phebe Olorunfemi, Miriam S. Udler, M.D., Ph.D.^1,3,7,8^ *Past Staff: Lizz Caulkins*

**Genetics Core – Baylor College of Medicine:** William Craigen, M.D., Ph.D.^3^, Hongzheng Dai, Ph.D., Shalini Jhangiani, Pengfei Liu, Ph.D.^3^, David Murdock, M.D, Jennifer E. Posey, M.D., Ph.D.^1,3,7,9^, Aniko Sabo, Ph.D.^3,4,7^, Eric Venner, Ph.D.^3^ *Past Staff: Lee-Jun Wong, Ph.D.*

**Central Laboratory – University of Florida:** William Winter, M.D.^4^, David Pittman^4^

**National Institutes of Diabetes and Digestive and Kidney Diseases:** Beena Akolkar, Ph.D. ^1,2,3,4,5,6,7,8,9^. *Past Staff: Christine Lee, M.D., M.S.*

**Other contributors:** David J. Carey, Ph.D.^8^, Geisinger Health System. Daniel Hood^8^, Regenstrief Institute. Santica M. Marcovina, Ph.D., Sc.D.^4,5,6,7^, Medpace Reference Laboratories. Christopher B. Newgard, Ph.D.^3,4^, Duke University Medical Center.

***Committees:***

^1^Adjudication, ^2^Ancillary Studies/Data Access, ^3^Discovery, ^4^Laboratory Implementation, ^5^Protocol Implementation, ^6^Protocol Oversight, ^7^Publications and Presentations, ^8^Recruitment and Retention, ^9^Steering
